# Advance Directives of German People with Parkinson's Disease Are Unspecific in regard to Typical Complications

**DOI:** 10.1155/2019/2107821

**Published:** 2019-08-04

**Authors:** Martin Klietz, Özlem Öcalan, Nils Schneider, Dirk Dressler, Stephanie Stiel, Florian Wegner

**Affiliations:** ^1^Department of Neurology, Hannover Medical School, Carl-Neuberg-Str. 1, 30625 Hannover, Germany; ^2^Institute for General Practice, Hannover Medical School, Carl-Neuberg-Str. 1, 30625 Hannover, Germany

## Abstract

Parkinson's disease (PD) is a progressive neurodegenerative movement disorder with an increased morbidity and mortality. People with PD (PwP) may suffer from decreased quality of life due to various motor and nonmotor symptoms. To a huge proportion, PwP have written an advance directive (AD); however, the content of these forms in regard to PD-specific complications is unclear. The aim of this study was to qualitatively and quantitatively analyze ADs of PwP in Germany. ADs of PwP were analyzed in a German sample of members of the German PD patient association. Participants completed a questionnaire about their AD and sent a copy of their AD to the study center for detailed analyses. ADs were qualitatively and quantitatively analyzed for general and PD-specific aspects and usefulness concerning treatment decisions. 82 PwP were included in the study, and in 76, an AD could be analyzed. Family members, notaries, lawyers, and general physicians mainly counseled writing of the ADs. 4 PwP consulted a neurologist to establish a specific AD for PD. In the analysis, ADs displayed a good specificity for general aspects, but they were unspecific to PD in the vast majority of cases. PwP should be encouraged to create an AD early in their disease and adapt it in the course of the disease. PD-specific aspects for an AD could be details in relation to dopaminergic therapies at the end of life, management of non-oral advanced therapies, neuropsychiatric symptoms, dementia, and swallowing disturbances.

## 1. Introduction

Parkinson's disease (PD) is the second most common neurodegenerative disease worldwide [1] with an estimated increase in prevalence during the next decades due to demographic change [2]. PD is a progressive movement disorder with an increased mortality compared to the general population [3]. Especially, aspiration pneumonia is significantly more often the cause of death in people with PD (PwP) compared to the general population [4–7]. Prior to the end-of-life stage, PwP suffer from various symptoms for a long time during the progression of the disease [8]. This time frame of suffering and decline of health-related quality of life is longer in PD compared to oncological diseases [9].

In the course of the disorder, PwP may suffer from disease-specific complications like akinetic crisis, cognitive decline, psychiatric symptoms, dysphagia, falls, and urinary symptoms [10]. These symptoms dramatically affect the quality of life of PwP in early and advanced stages [11, 12]. In later disease stages, palliative care interventions may be implemented to sustain or even improve health-related quality of life in PwP [13, 14].

Advance directives (ADs) are legal documents in which people state their will or specify in advance what actions should be engaged if they are no longer capable of decision-making because of disease or disability. This written living will is immediate and binding on doctors, nurses, and other stakeholders, regardless of the nature or stage of the person's disease. Patients may use standard forms provided by several institutions or write their own advance directive. The AD will become valid with the indication of the place, date, and a handwritten signature. An AD may be amended or revoked at any time. However, advice of a lawyer or a notary is not obligatory for the legal validity of an advance directive. In 2016, the German federal court ruled that ADs have to be specific in regard to treatment wishes; otherwise, the document is not legally valid and the healthcare professionals are not bound to the content of the directive. One recent study of our group was able to demonstrate that a majority of advanced PwP (69.7%) had an advance directive [12]. However, the specificity for PD of these ADs is yet unknown [4].

This exploratory study aims to qualitatively and quantitatively analyze ADs in a cohort of German PwP. How specific are these ADs for disease-specific complications in PD and who counseled the patients in which amount of time? Results should help patients to create and healthcare professionals to better and specific counseling ADs for PwP.

## 2. Methods

### 2.1. Participants

We obtained approval from the Local Ethics Committee of Hannover Medical School (No. 3123-2016, Amendment 2018), and patients gave written informed consent. All experiments were in accordance with the guidelines and regulations of the local ethics committee, the Declaration of Helsinki, and European data protection laws. Our sample included 82 PwP from all over Germany. Between May and October 2018, PwP were recruited from our movement disorder outpatient clinic, our neurological wards, and German PD support groups. The study questionnaire was handed to local patients and mailed to nonlocal patients (equal to recruitment in [15]). Inclusion criteria for PwP were defined as neurologically confirmed diagnosis of idiopathic PD and disease duration of at least 1 year. PwP suffering from dementia or atypical parkinsonism were excluded from this analysis. Patients without AD were not able to participate in our study.

### 2.2. Measures

All PwP got a self-assessment questionnaire specially constructed to assess aspects concerning their disorder (e.g., disease duration, self-estimated Hoehn and Yahr stage in the best medical on) and AD (e.g., time point and motivation of writing and location of deposit). PwP had to send the written consent, completed questionnaire, and a copy of their AD to the study center.

The second part of the analysis evaluated general formal characteristics, design features, and content specificity of the ADs for PD in relation to defined complications like akinetic crisis, cognitive decline, psychiatric symptoms, dysphagia, and urinary symptoms. Additionally, particular therapies specified in an AD were analyzed like treatment on an intensive care medicine, mechanical ventilation, and antibiotic treatment of severe life-threatening infections. PD-specific and general characteristics were analyzed using traffic light coloring. Hence, because this is the first qualitative analysis of ADs of PwP, we had to choose a more holistic approach, since we did not want to quantify agreement or refusal to a specific intervention. However, we want to quantify how specific and by this clinically useful PwP express their wishes concerning general and disease-specific complications. This traffic light approach appeared to be the most fitting way of quantification of these qualitative results. Red indicated no notification of the specific item. Yellow indicated an unspecific notification of a defined item without clear wishes addressing the treating physician. Green indicated a clear and specific wish of the patients concerning a specific item.

### 2.3. Statistics

Statistics were carried out using SPSS 25.0 (IBM, Armonk, NY) and Prism 5.00 (San Diego, California, USA). Qualitative data were analyzed with MAXQDA using summarizing content analysis.

## 3. Results

### 3.1. Patient Characteristics and Disease-Related Information

In total, we contacted 186 PwP from PD support groups or via the department of neurology. During data collection, 82 PwP were enrolled; this equates a response rate of 44.1%. Six of them missed to send their AD but answered the self-completed questionnaire, and another patient did not deliver the self-completed questionnaire but send his AD. The study sample is in average 71.2 years old (range 47–87) with 41.5% females. The majority of participants are married/living in a relationship (82.9%) with a high (47.6%) or middle (29.3%) level of education (Table 1).

The PD course of the participants lasted on average for 8.8 years (range 1–26 years). The study sample covered all five Hoehn and Yahr stages of PD, mainly consisting of stages 3 (54.9%) to 4 (26.8%) (Table 2).

### 3.2. Patients' Self-Assessment of Advance Directives

The received ADs were created in average 6.2 years before the study begins (range 0–38 years) (Table 3). The most common reasons of patients' for writing ADs were as follows: (i) the expression of autonomy and taking responsibility for end-of-life care (*n* = 30), (ii) patients' own (*n* = 16) or witnessed disease and care experiences (*n* = 15), (iii) to inform and protect each other in the family (*n* = 5), or (iv) influence of media (*n* = 3), legal regulations (*n* = 2), or advice from other persons (*n* = 2).

The majority of ADs was dated (90.3%) and contained personal patient data such as the full name (89.0%) or address (71.9%). Most ADs were based on predefined formats of official documents (54.9%); another 37.8% were freely worded letters. Aids from the German Parkinson Association were used by 4.9% of the participants when developing their AD. The majority of participants had advice from their wife/husband/partner (80.5%), other family members (34.1%), or friends (20.7%). Four patients were counseled on their AD by a neurologist (Table 3).

20 patients (24.4%) made changes to the initial AD version in average 6.8 years (range 0.25–13 years) after the first draft (Table 3). The most frequent motivations for making changes were (i) content-related modifications due to new experiences, worsening of PD, or changes of living will (*n* = 8), (ii) formal adaptions to new legal regulations (*n* = 4), and (iii) modifications of personal data such as the address (*n* = 4). According to the requirements of the legislation, all but one patient signed their AD. In 21.9% of the ADs, a wife/husband/partner and in 14.6% a family physician cosigned the patients' AD. Most often the AD was deposited at the patients' homes (92.7%), additionally at the family physician's practice (32.9%) or at a family member's home (31.7%) (Table 3).

Almost all participants named one or more healthcare proxies (95.1%) in their AD, who were most often wife/husband/partner (82.1%) or child/children (64.1%). Patients assessed the intensity of previous conversations with proxies as extensive (75.6%) or brief (23.1%). Only one patient reported of no conversation with his proxy about the AD (Table 3).

### 3.3. Analysis of Advance Directives in the Study Center

A minority of ADs considered patients' personal biographical aspects (2.4%), used free texts in general (29.3%) or in particular about personal norms and values (9.8%), or messages to other persons (19.5%). Included in the patients' ADs were contact details of trusted physicians in 39%, doctors' confirmations of advising the patients, and giving medical information in 9.8%. Notes on the AD duration or time point until when the AD is valid were included in 36.6%, resignatures to confirm validity in 24.4%, and notes on legal validity in 64.6%. Notes on wishes for spiritual support were integrated in 34.2% and a documented will concerning organ donation in 53.7%. At least 7.3% of the participants state in their self-assessment that they considered PD-specific aspects in their AD (Table 4).

**Figure 1:**
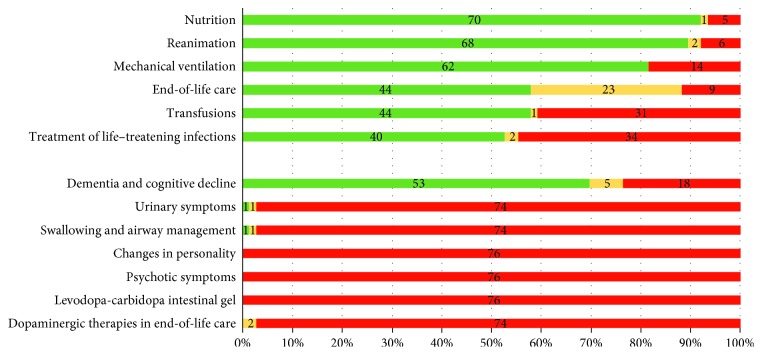
Analysis of general and specific aspects of the advance directives (ADs) of 76 people with Parkinson's disease. Green color indicates the amount of clear and specific wishes of the patients concerning individual aspects of their AD. Yellow indicates the amount of mentioned but unspecific wishes of the patients in relation to individual aspects of the AD with no clear guideline for the clinical management. Red indicates the amount of neglected aspects. Abbreviations: PD, Parkinson's disease; LCIG, levodopa-carbidopa intestinal gel.

Applying the traffic light colors to assess general healthcare issues as well as PD-specific aspects in the ADs, it became apparent that a considerable proportion of ADs contained clear and specific statements (green light) on general healthcare issues such as nutrition (92.1%), reanimation (89.5%), mechanical ventilation (81.6%), end-of-life care (57.9%), transfusions (57.9%), and treatment of life-threatening infections (52.6%). On the contrary, PD-specific aspects such as jejunal levodopa intestinal gel infusion, complications like psychotic symptoms, and changes in personality were not considered (red). Only two patients mentioned dopaminergic therapies in end-of-life care, but these wishes were very unspecific (yellow). Some clear and specific wishes in handling PD-related dementia and cognitive decline (69.7%), urinary symptoms (1.3%), and swallowing and airway management (each 1.3%) (green) were found (Figure 1).

## 4. Discussion

ADs are legal documents for decision-making if the patient is not able to state his or her current will. In German federal law, ADs have to state specific wishes of the patients to be a valid guideline for treatment decisions when the patient is not able to decide. However, at the beginning of our study, it was unclear how specific ADs of PwP are in relation to disease-specific complications.

In the present study, we evaluated ADs of German PwP in a qualitative and quantitative manner to analyze counseling and relevance for clinical decision-making. First, we recognized all except for one included ADs as legal documents by German federal law. Recruited PwP were in all stages of PD, mostly Hoehn and Yahr stage 3. Family physicians, lawyers, or notaries mainly counseled patients' writing of ADs. Analyzing the ADs for general and PD-specific issues (Figure 1), we found a good guidance for general aspects of end-of-life care preferences independent from an underlying disease. In contrast, only a minority of ADs mentioned PD-specific issues including clear treatment wishes which are of great importance for this particular patient group. Probably, most PwP are not aware of these possibilities and do not mention PD-specific aspects in their AD. Neurologists advised PD patients only in a minority of cases. Counseling by a neurologist might improve the unspecific ADs of PwP by raising awareness for specific complications and treatment possibilities in the course of the disease. It is unclear why our participants did not use the assistance of neurologists for AD creation. We could speculate that rare appointments, lack of perception, and immediate need for a legally valid AD might be important factors. Furthermore, the rare usage of neurological expertise might be due to a neglect for advanced PwP [12] and lack of clear recommendations for AD creation in PD. In a local cohort of advanced German PD patients, only half of the patients manage to schedule regular appointments by a neurologist [12]. However, it remains unclear if general neurologists are experienced to counsel AD creation for PwP. Palliative care is an emerging field in neurology; nevertheless, the proportion of neurologists with palliative care and end-of-life care qualifications is not known and seems to be small but increasing (personal estimation of the authors). Structured guidelines should help the neurologists to better advise AD creation in the future.

The vast majority of the investigated ADs were standard forms with only limited possibilities for individualization (Table 1). Only a minor part of our analyzed ADs contained individual elements like a short biography, own cultural norms, or a message to relatives. By using standard forms, patients find only a mild or moderate possibility for individualization. However, these individual parts can be of fundamental importance for the clinician and relatives to develop a better understanding for the needs and wishes of the patient. Some patients feel insecure about the legal relevance of their AD which might be a possible reason for the frequent usage of standard documents (personal communication with our patients) [16]. In these documents, the patients already find the most important features for the conception of a general AD. These ADs should be specific for some general aspects that occur frequently in an end-of-life setting, for example, cardiopulmonary reanimation, mechanical ventilation, treatment on an intensive care unit, analgesia, or antibiotic therapy of a life-threatening infection.

PD-specific aspects were missing in most AD forms including the guideline of the German PD association that gives only unspecific recommendations. However, PwP may undergo specific complications in the course of the disease [9, 16]. To our knowledge, PD-specific issues have never been summarized in the context of palliative care decision-making. Interestingly, Seitzer et al. described the full spectrum of ethical problems and decision-making at the example of amyotrophic lateral sclerosis [17]. In the following passages, the most relevant PD-specific complications are discussed in detail.

Treatment with dopaminergic medication is extremely important to reduce unpleasant symptoms like painful rigidity or tremor and prevent akinetic crisis in PD end-of-life care. General or internal medicine physicians might not be similarly aware of PD and the treatment possibilities as neurologists. In this case, a specific passage related to PD medical therapy in the AD would be helpful for the clinician and could improve treatment. In our cohort, only one patient inserted a similar specific passage in the AD. At the time of diagnosis, but latest at the beginning of advanced PD, this aspect should be mentioned and noted in the AD.

Neuropsychiatric symptoms like hallucinations, anxiety, depression, and impulse control disorders might complicate the course of PD. These symptoms might be related or unrelated to PD treatment and impact the quality of life of the patient in an extensive manner. We found no specific AD modifications related to neuropsychiatric symptoms in PD. Before or by the first occurrence of these symptoms, the patient might want to add a relevant passage to the AD, e.g., the wish to be referred to a neuropsychiatric specialist. The patient could also mention aspects of polypharmacotherapy and drug safety in the AD, for instance, that symptom control should be more important than drug safety aspects especially in the palliative intervention phase [12, 18].

The number of patients suffering from dementia will dramatically increase in the next decades by demographic change [19, 20]. Since dementia has a high prevalence in western countries, a huge proportion of PwP also mentioned it in their AD. However, aspects of dementia are also very unspecific and concepts for specific ADs are still under development [21]. Specific ADs for dementia would help the healthcare proxies in decision-making and would reduce anxiety of surrogate decision-making [22]. Gaster et al. suggested to include changes in cognition and other milestones of disease progression as a marker for change of treatment goals in a dementia-specific AD [21]. PD and dementia share some common aspects, but the management diverse in certain aspects of the diseases [23]. Especially in the late stages of PD, cognitive deficits and dementia are a common condition [12, 24]. However, the corresponding passages of the ADs were not specific to PD-related dementia. It is desirable to generate a specific AD before the development of cognitive impairments in PwP [25], because the identification of patient wishes will be more difficult or even impossible with the progression of cognitive decline to dementia.

In advanced PD, non-oral rescue therapies like deep brain stimulation, apomorphine s.c. pump, and levodopa-carbidopa intestinal gel (LCIG) pump are available. These 3 therapies have a certain profile of adverse effects. An informed patient could define the start or stop criteria for a specific therapy. In the case of deep brain stimulation, a patient can refuse to undergo an operative change or a recharging of the stimulator if the battery is exhausted and the physician judges treatment efficacy as low. A specific feature of LCIG therapy is the possibility of additional nutrition of the patient via the gastric line. The patient under LCIG therapy could state if he wants to receive nutrition or water supplementation, or rather refuse this therapy in an end-of-life setting [26].

Another difficult issue is the integration of the caregivers, who are in most of the cases, also the healthcare proxies, in the process of decision-making. Actual German data indicate a moderate burden in caregivers of advanced stage PwP [15, 27].

### 4.1. Limitations

Data from our study give an overview about a large number of legally valid ADs from German PwP. Nevertheless, this analysis cannot assure to have covered the entire spectrum of possible AD contents in PD. This study was not designed to analyze ADs of PwP under advanced therapies, so we did not quantify the number of patients under deep brain stimulation or pump therapies. The number of patients under non-oral therapies with specific wishes concerning these therapies might be underestimated for this reason. However, our data were in line with our clinical experience. Some of these data might be specific for the German health system and are not fully transferable to countries with other legal concepts concerning ADs. Patient recruitment from PD support groups might have led to a mild selection bias, because we included patients with more support in general, a larger network of specialists, and higher interest in this area. However, in our study, we had a very good response rate of 44.1% of the contacted 186 PwP but cannot rule that people with generally higher interest in ADs might have answered the questionnaires more often.

## 5. Conclusion

PwP should be encouraged to create an AD early in the course of the disease, so that the capacity of decision-making will not be disturbed by their severe motor or nonmotor symptoms [28–30]. If new PD-related aspects emerge, the patient should be counseled by a neurological specialist to modify the AD specifically and communicate these wishes to the healthcare proxy. Specific scenarios of disease complications might help PwP to address their wishes concerning advance care planning [31].

## Figures and Tables

**Table 1:** Demographic data on study sample (*N* = 82).

Item	Answer option	*N* = 82
*Demographic data*		
Sex	Female	34 (41.5%)
	Male	47 (57.3%)
	Missing data	1 (1.2%)

Age (years)	Mean ± SD	71.2 ± 9.9
	Range	47–87
	Missing data	3 (3.7%)

Marital status	Married	68 (82.9%)
	Widowed	8 (9.8%)
	Divorced/living apart	4 (4.9%)
	Single	1 (1.2%)
	Missing data	1 (1.2%)

Educational level	High	39 (47.6%)
	Middle	24 (29.3%)
	Low	18 (21.9%)
	Missing data	1 (1.2%)

SD, standard deviation; PD, Parkinson's disease; H&Y, Hoehn and Yahr stage.

**Table 2:** Disease-related data on study sample (*N* = 82).

Item	Answer option	*N* = 82
*Disease-related data*		
Time since diagnosis	0–5 years	26 (31.7%)
	5–10 years	31 (37.8%)
	>10 years	23 (28.1%)
	Missing data	2 (2.4%)

Duration of PD course since diagnosis (years)	Mean ± SD	8.8 ± 5.7
	Range	1–26
	Missing data	2 (2.4%)

Time since symptom onset (years)	Mean ± SD	10.6 ± 6.4
	Range	2–30
	Missing data	5 (6.1%)

Current H&Y stage of disease	1	7 (8.5%)
	2	1 (1.2%)
	3	45 (54.9%)
	4	22 (26.8%)
	5	4 (4.9%)
	Missing data	3 (3.7%)

SD, standard deviation; PD, Parkinson's disease; H&Y, Hoehn and Yahr stage.

**Table 3:** Patient-assessed information about writing of ADs (*N* = 82).

Item	Answer option	*N* = 82
Time since AD creation (years)	Mean	6.2 ± 5.4
	Range	0–38
	Missing data	4 (4.9%)

AD is dated	Yes	74 (90.3%)
	No	2 (2.4%)
	Missing data	6 (7.3%)

AD contains personal data of the patient	Full name	73 (89.0%)
	Postal address	59 (71.9%)
	Contact details	6 (7.3%)
	No personal data	6 (7.3%)

Use of aids	From German Parkinson Association	4 (4.9%)

Format used in AD	Letter	31 (37.8%)
	Official document	45 (54.9%)
	Missing data	6 (7.3%)

Advice from (multiple answers possible)	Wife/husband/partner	66 (80.5%)
	Notary/lawyer	29 (35.4%)
	Family members (other than spouse)	28 (34.1%)
	Family physician	25 (30.5%)
	Friends	17 (20.7%)
	Patient support group	7 (8.5%)
	Neurologist	4 (4.9%)
	Religious counsellor	3 (3.6%)
	Others	6 (7.3%)

Healthcare proxies named in AD	Yes	78 (95.1%)
	No	3 (3.7%)
	Missing data	1 (1.2%)

Healthcare proxies relationship to patient (multiple answers possible)	Wife/husband/partner	64 (82.1%)
	Children	50 (64.1%)
	Friends	3 (3.9%)
	Family members	1 (1.3%)
	Others	3 (3.9%)

Intensity of previous conversation with proxy (*n* = 78)	Extensive conversations	59 (75.6%)
	Brief conversations	18 (23.1%)
	No conversations	1 (1.3%)

AD signed by (multiple answers possible)	Patient	81 (98.8%)
	Wife/husband/partner	18 (21.9%)
	Family physician	12 (14.6%)
	Children	9 (11.0%)
	Notary	5 (6.1%)
	Friends	2 (2.4%)
	Lawyer	1 (1.2%)
	Other persons	2 (2.4%)
	Missing data	1 (1.2%)

Location of deposit (multiple answers possible)	Patients' homes	76 (92.7%)
	Family physician's practice	27 (32.9%)
	Family member's home	26 (31.7%)
	Chamber of notaries/central registers	7 (8.5%)
	Notary/lawyer	7 (8.5%)
	Hospital/special outpatient clinic	3 (3.7%)
	Friend's home	3 (3.7%)
	Neurologist's practice	2 (2.4%)
	Other locations	4 (4.9%)
	Missing data	1 (1.2%)

Changes made since initial version	Yes	20 (24.4%)
	No	61 (74.4%)
	Missing data	1 (1.2%)

Time between initial version and changes (years) (*n* = 20)	Mean	6.8 ± 4.6
	Range	0.25–13
	Missing data	3 (15.0%)

SD, standard deviation; PD, Parkinson's disease; AD, advance directive.

**Table 4:** Contents of the ADs (*N* = 82).

Item	Answer option	*N* = 82
Individual free texts	Yes	24 (29.3%)
	No	52 (63.4%)
	Missing data	6 (7.3%)

Personal patient biography	Yes	2 (2.4%)
	No	74 (90.3%)
	Missing data	6 (7.3%)

Free texts about norms and values	Yes	8 (9.8%)
	No	68 (82.9%)
	Missing data	6 (7.3%)

Messages/advice to particular persons	Yes	16 (19.5%)
	No	60 (73.2%)
	Missing data	6 (7.3%)

Contact details of trusted physician	Yes	32 (39.0%)
	No	44 (53.7%)
	Missing data	6 (7.3%)

A doctor confirms advising the patient and giving medical information	Yes	8 (9.8%)
	No	68 (82.9%)
	Missing data	6 (7.3%)

Patient confirms getting medical information	Yes	16 (19.5%)
	No	60 (73.2%)
	Missing data	6 (7.3%)

Confirms the patients' ability to consent/to make a will (multiple answers possible)	Yes, by a physician	10 (12.2%)
	Yes, by other trusted persons	8 (9.8%)
	Yes, by a notary	7 (8.5%)
	Yes, by a lawyer	2 (2.4%)
	No	58 (70.7%)
	Missing data	6 (7.3%)

Includes notes on (multiple answers possible)	Duration/time point of validity	30 (36.6%)
	Revocation	44 (53.7%)
	Changes	48 (58.5%)

Includes note on legal validity of AD	Yes	53 (64.6%)
	No	23 (28.1%)
	Missing data	6 (7.3%)

Includes confirmation of validity of AD by re-signing	Yes	20 (24.4%)
	No	56 (68.3%)
	Missing data	6 (7.3%)

States will on organ donation	Consent	24 (29.3%)
	Rejection	20 (24.4%)
	Missing data	38 (46.3%)

Includes note on wishes for spiritual support	Yes	28 (34.2%)
	No	48 (58.5%)
	Missing data	6 (7.3%)

Considers specific aspects of PD (self-assessed)	Yes	6 (7.3%)
	No	75 (91.5%)
	Missing data	1 (1.2%)

PD, Parkinson's disease; AD, advance directive.

## Data Availability

Due to the sensitive nature of the data and the related questions asked in our study, participants were assured raw data would remain confidential and would not be shared.
